# Central Nervous System Idiopathic Inflammatory Demyelinating Disorders in South Americans: A Descriptive, Multicenter, Cross-Sectional Study

**DOI:** 10.1371/journal.pone.0127757

**Published:** 2015-07-29

**Authors:** Regina Maria Papais-Alvarenga, Claudia Cristina Ferreira Vasconcelos, Adriana Carra, Ibis Soto de Castillo, Sara Florentin, Fernando Hamuy Diaz de Bedoya, Raul Mandler, Luiza Campanella de Siervi, Maria Lúcia Vellutini Pimentel, Marina Papais Alvarenga, Marcos Papais Alvarenga, Anderson Kuntz Grzesiuk, Ana Beatriz Calmon Gama Pereira, Antonio Pereira Gomes Neto, Carolina Velasquez, Carlos Soublette, Cynthia Veronica Fleitas, Denise Sisteroli Diniz, Elizabeth Armas, Elizabeth Batista, Freda Hernandez, Fernanda Ferreira Chaves da Costa Pereira, Heloise Helena Siqueira, Hideraldo Cabeça, Jose Sanchez, Joseph Bruno Bidin Brooks, Marcus Vinicius Gonçalves, Maria Cristina Del Negro Barroso, Maria Elena Ravelo, Maria Carlota Castillo, Maria Lúcia Brito Ferreira, Maria Sheila Guimarães Rocha, Monica Koncke Fiuza Parolin, Omaira Molina, Patricia Beatriz Christino Marinho, Paulo Pereira Christo, Renata Brant de Souza, Silvio Pessanha Neto, Solange Maria das Graças Camargo, Suzana Costa Machado, Vanderson Carvalho Neri, Yara Dadalti Fragoso, Helcio Alvarenga, Luiz Claudio Santos Thuler

**Affiliations:** 1 Department of Neurology, Universidade Federal do Estado do Rio de Janeiro, Rio de Janeiro, Brazil; 2 Department of Neurology, Hospital Federal da Lagoa, Ministério da Saúde, Rio de Janeiro, Brazil; 3 Clinica de Neuroimunologia, Rio de Janeiro, Brazil; 4 Department of Neurology, Hospital Británico, Buenos Aires, Argentina; 5 Department of Neurology, Hospital Clínico de Maracaibo, Maracaibo, Venezuela; 6 Department of Neurology, Instituto de Prevision Social, Asunción, Paraguay; 7 Department of Neurology, Universidad Unida, Asunción, Paraguay; 8 Department of Neurology, John Hopkins, Washington, District of Columbia, United States of America; 9 Department of Neurology, Santa Casa da Misericórdia, Rio de Janeiro, Brazil; 10 Department of Neurology, Rede Sarah de Reabilitação, Rio de Janeiro, Brazil; 11 Department of Neurology, Centro de Reabilitação Integral Dom Aquino Corrêa, Mato Grosso, Brazil; 12 Department of Neurology, Universidade Severino Sombra, Vassouras, Rio de Janeiro, Brazil; 13 Department of Neurology, Santa Casa da Misericórdia, Belo Horizonte, Minas Gerais, Brazil; 14 Department of Neurology, Hospital Universitário, Caracas, Venezuela; 15 Department of Neurology, Hospital das Clínicas da Universidade Federal de Goiás, Goiania, Brazil; 16 Neuroclinica, Volta Redonda, Rio de Janeiro, Brazil; 17 Department of Neurology, Universidade Federal de Mato Grosso, Cuiabá, Brazil; 18 Department of Neurology, Hospital Ofir Loiola, Pará, Brazil; 19 Department of Neurology, Centro de Referência em Esclerose Múltipla do Litoral Paulista, São Paulo, Brazil; 20 Department of Neurology, Joinville, Santa Catarina, Brazil; 21 Department of Neurology, Hospital JM de los Rios, Caracas, Venezuela; 22 Department of Neurology, Hospital da Restauração, Recife, Pernambuco, Brazil; 23 Department of Neurology, Hospital Santa Marcelina, São Paulo, Brazil; 24 Clínica neurológica, Paraná, Brazil; 25 Department of Neurology, Imperial Hospital de Caridade, Florianópolis, Santa Catarina, Brazil; 26 Clinical Research Division Instituto Nacional de Câncer, Rio de Janeiro, Brazil; Medical University of Innsbruck, AUSTRIA

## Abstract

The idiopathic inflammatory demyelinating disease (IIDD) spectrum has been investigated among different populations, and the results have indicated a low relative frequency of neuromyelitis optica (NMO) among multiple sclerosis (MS) cases in whites (1.2%-1.5%), increasing in Mestizos (8%) and Africans (15.4%-27.5%) living in areas of low MS prevalence. South America (SA) was colonized by Europeans from the Iberian Peninsula, and their miscegenation with natives and Africans slaves resulted in significant racial mixing. The current study analyzed the IIDD spectrum in SA after accounting for the ethnic heterogeneity of its population. A cross-sectional multicenter study was performed. Only individuals followed in 2011 with a confirmed diagnosis of IIDD using new diagnostic criteria were considered eligible. Patients’ demographic, clinical and laboratory data were collected. In all, 1,917 individuals from 22 MS centers were included (73.7% female, 63.0% white, 28.0% African, 7.0% Mestizo, and 0.2% Asian). The main disease categories and their associated frequencies were MS (76.9%), NMO (11.8%), other NMO syndromes (6.5%), CIS (3.5%), ADEM (1.0%), and acute encephalopathy (0.4%). Females predominated in all main categories. The white ethnicity also predominated, except in NMO. Except in ADEM, the disease onset occurred between 20 and 39 years old, early onset in 8.2% of all cases, and late onset occurred in 8.9%. The long-term morbidity after a mean disease time of 9.28±7.7 years was characterized by mild disability in all categories except in NMO, which was scored as moderate. Disease time among those with MS was positively correlated with the expanded disability status scale (EDSS) score (r=0.374; p=<0.001). This correlation was not observed in people with NMO or those with other NMO spectrum disorders (NMOSDs). Among patients with NMO, 83.2% showed a relapsing-remitting course, and 16.8% showed a monophasic course. The NMO-IgG antibody tested using indirect immunofluorescence (IIF) with a composite substrate of mouse tissues in 200 NMOSD cases was positive in people with NMO (95/162; 58.6%), longitudinally extensive transverse myelitis (10/30; 33.3%) and bilateral or recurrent optic neuritis (8/8; 100%). No association of NMO-IgG antibody positivity was found with gender, age at onset, ethnicity, early or late onset forms, disease course, or long-term severe disability. The relative frequency of NMO among relapsing-remitting MS (RRMS) + NMO cases in SA was 14.0%. Despite the high degree of miscegenation found in SA, MS affects three quarters of all patients with IIDD, mainly white young women who share similar clinical characteristics to those in Western populations in the northern hemisphere, with the exception of ethnicity; approximately one-third of all cases occur among non-white individuals. At the last assessment, the majority of RRMS patients showed mild disability, and the risk for secondary progression was significantly superior among those of African ethnicity. NMO comprises 11.8% of all IIDD cases in SA, affecting mostly young African-Brazilian women, evolving with a recurrent course and causing moderate or severe disability in both ethnic groups. The South-North gradient with increasing NMO and non-white individuals from Argentina, Paraguay, Brazil and Venezuela confirmed previous studies showing a higher frequency of NMO among non-white populations.

## Introduction

Multiple sclerosis (MS) has been considered a rare disease in South America (SA). The environmental and genetic factors of its extensive tropical territory and significant racial heterogeneity might protect SA against the disease. Moreover, the lack of scientific publications regarding MS in SA prior to the 1990s led to the misconception that a low prevalence of MS (<5/100,000) existed throughout the region compared with the distribution of MS worldwide [1] Thus, all of the information regarding the natural history of MS was based on studies of white people in the northern hemisphere, where the prevalence of MS is high [2,3]. Over the past 20 years, the knowledge regarding the prevalence, clinical course, and risk factors associated with the development of MS in SA has widely expanded [4]. The major factors that promoted this scientific growth were the incorporation of magnetic resonance imagining (MRI) as a tool to diagnose idiopathic inflammatory demyelinating diseases (IIDDs), the availability of the Internet (which has connected health science professionals and investigators across the world), and the Food and Drug Administration (FDA) approval of immunomodulatory drugs (expensive medications that began to be distributed free of charge by public health systems through MS treatment reference centers organized across various SA countries) [5].

A major development over the past two decades was the recognition of neuromyelitis optica (NMO; i.e., Devic’s syndrome) as a special form of neuro inflammatory disorder of the central nervous system (CNS). NMO has unique clinical, imaging, laboratory, and pathologic characteristics as well as a pathogenic mechanism that distinguishes it from MS. Mandler *et al*. [6] demonstrated normal cranial MRI scans, the absence of IgG oligoclonal bands (OCBs), and (most importantly) a characteristic and distinct neuropathology in a seminal study of eight women of different ethnicities (four whites, three Latinas, and one African) in New Mexico, USA. These women presented with severe and selective involvement of the optic nerve and spinal cord with a poor prognosis. No lesions were observed in the brain MRI scans, whereas the spinal cord MRI scans demonstrated long, cavitated, and enhanced lesions of more than three segments. Characteristic thick blood vessels with perivascular necrosis were found in the CNS, with long necrotic lesions in the spinal cord. These findings led to the hypothesis that this autoimmune disease was mediated by a soluble antibody. Those features were identified, confirmed, and expanded; moreover, they were included in the NMO diagnostic criteria to distinguish it from MS [7].

The identification of an autoantibody, NMO-IgG, with high specificity for NMO that was also present in partial NMO syndromes represented a milestone in the study of IIDD. In the original study applied indirect immunofluorescence (IIF) with a composite substrate of mouse tissues and observed a high frequency of antibody positivity in patients with NMO; less antibody positivity was observed in patients with partial syndromes with a high risk of conversion to NMO: longitudinally extensive transverse myelitis (LETM) and bilateral or recurrent optic neuritis (BRON). The antibody was also found in patients with optic spinal multiple sclerosis (OS-MS) Asian type and in rare cases of MS; it was not detected in other neurological diseases. This antibody was the first biological marker found in patients with IIDD [8]. Later, NMO-IgG was shown to bind selectively to the aquaporin 4 (AQP4) water channel, a transmembrane protein located in the astrocytic foot processes at the blood-brain barrier [9]. Brain MRI scans of NMO-IgG-positive individuals with NMO demonstrated lesions in the brainstem periaqueductal area, diencephalon, and periventricular areas that are characterized by high AQP4 expression [10]. Thus, the presence of neurological signs outside of the optic nerve and spinal cord, particularly those in the brainstem and brain, was verified. Positivity to the anti-AQP4 IgG antibody was included among the laboratory criteria for NMO in 2006 [11]. The term "NMO spectrum disorders" (NMOSDs) was coined to encompass NMO and other CNS inflammatory diseases in which the NMO-IgG antibody was identified. The clinical and laboratory features of NMOSD differ from those found in MS [12].

The distribution of IIDD according to the new NMO and MS criteria has been investigated with regard to different populations. The results indicated the following relative frequencies of NMO cases among patients with MS: 1.2% in Australia [13], 1.5% in Italy [14], 8% in Mexico [15], 15.2% in southeastern Brazil [16], and 27% in Central America [17]. The highest frequency of cases featuring the selective involvement of the optic nerve and spinal cord s found in Japan, where 33% of patients with MS have Asian-type OSMS [18]. The analysis of these studies suggests that many people have NMO in non-white populations who live in geographic areas with low rates of MS.

SA was colonized by Europeans from the Iberian Peninsula (Spain and Portugal), and their miscegenation with natives and African slaves resulted in significant racial heterogeneity. More than 50% of Brazil is of African descent [19]. In the SA countries along the Pacific Coast, the admixture of whites and natives gave rise to the so-called Mestizos. This study analyzed the IIDD spectrum in SA after accounting for the ethnic heterogeneity of its population.

## Material and Methods

This descriptive, multicenter, and cross-sectional study was approved by the Human Research Ethics Committee of the Hospital Universitário Gaffrée e Guinle at the Universidade Federal do Estado do Rio de Janeiro (number # 243.375, April 9, 2013)

We collected data from 21 SA MS centers, and all of the data were handled anonymously (no individual could be identified). Individual information was stored and treated in accordance with ethical regulations (S1 File). This study design did not require informed patient consent. We included data from individuals with IIDD who were followed regularly from January to December 2011. Coordinators (LCST, RMPA) invited neurologists from reference centers for the treatment of demyelinating diseases in SA to participate in the study in July 2012.

### Inclusion of patients

Only individuals followed at the participating centers in 2011 (either old or new cases) with a confirmed diagnosis of IIDD based on the current criteria were considered eligible. The tests required for an IIDD diagnosis included the following: brain and spinal cord MRI, cerebrospinal fluid (CSF) analysis including measurement of the IgG index or OCB analysis and NMO-IgG antibody testing [20].

### IIDD phenotypes

Patients with IIDD were classified and analyzed with regard to the following six major categories and their subcategories.

[1] Acute IIDD with encephalopathy: [1.1] pseudotumoral-acute and monophasic forms of IIDD with extensive focal lesions based on brain MRI (unrelated to MS or NMO); [1.2] Marburg-acute and monophasic forms of IIDD associated with extensive multifocal lesions based on brain MRI; [1.3] Balo’s concentric sclerosis-acute monophasic or relapsing IIDD associated with extensive concentric brain lesions based on MRI [20]

[2] ADEM: [2.1] monophasic-acute episode possibly preceded by infection or vaccination characterized by multifocal cerebral manifestations accompanied by a change in consciousness and or mental confusion associated with acute inflammatory lesions based on MRI, diffuse and symmetrical; [2.2] recurrence of typical symptoms of ADEM after 3 months of the initial episode without new lesions based on MRI [20]

[3] Clinically isolated syndrome (CIS): suggestive of an MS diagnosis not meeting the following radiological criteria for MS: [3.1] monofocal (unilateral optic neuritis, internuclear ophthalmoplegia, or other brainstem isolated syndromes, partial myelopathy) or [3.2] multifocal CIS [21]

[4] MS and its clinical course patterns: [4.1] relapsing remitting (RR) at onset and [4.2] primary progression (PP), fulfilling the McDonald criteria revision [22]

[5] NMO: [5.1] monophasic or [5.2] recurrent, with selective (but not exclusive) focal involvement of the optic nerve and spinal cord; diagnosis requires ON plus TM and two of the following three laboratorial criteria: normal brain MRI or no suggestion of MS, extensive spinal cord MRI lesion (≥3 vertebral segments), and positive NMO-IgG antibody [11]

[6] Other NMO syndromes: ***[6*.*1] limited NMO syndromes*** [12] including [6.1.1] monophasic transverse myelitis with LETM (≥3 vertebral segment spinal cord lesion observed on MRI scan); [6.1.2] recurrent LETM [6.1.3]; LETM associated with brain lesions typical of NMO (hypothalamic, corpus callosal, periventricular, or brainstem); [6.1.4] ON associated with brain lesions typical of NMO (hypothalamic, corpus callosal, periventricular, or brainstem); [6.1.5] BRON; ***[6*.*2] Asian OS-MS*** [18], recurrent ON and TM not meeting NMO criteria (2006) [11]

### Exclusion criteria

Patients still under investigation, those not meeting the criteria for diagnosis, those who attended the neurology service solely to obtain a second opinion, and those who lived in cities outside the location of the treatment center were excluded. Registries without a specific diagnosis were also excluded from the analysis.

### Data collection

The information collected included the following: identification (name of participating neurologists, type of unit, town, and country); patient demographic and clinical data (name, initials, birth date, town/country of residence, year and age at onset of IIDD [early onset, i.e., pediatric MS: <18 years; late onset: ≥50 years], gender, ethnicity/skin color [white and non-white, i.e., Mestizo (mixed white and American Indian ancestry) African (including mixed white and black ancestry) Asian (including mixed white and Asian ancestry)]), and score on the Expanded Disability Status Scale (EDSS) [23]. The disability classification according to the EDSS is 0–2.5 = mild, 3.0–5.5 = moderate, and ≥6 = severe. The multiple sclerosis severity score (MSSS) recorded at the last assessment was only applied in patients with MS. The MSSS is an index that adjusts the EDSS score based on the time of the disease (1–30 years). The MSSS varies from 0.01 to 9.99. [24] The severity of disease according to the MSSS was classified into four categories: benign<0.45, mild-moderate = 0.46–5.00, advanced-accelerated = 5.00–8.23), and aggressive-malignant>8.24 [25]. Information was collected using the laboratory method applied to detect the NMO-IgG antibody and seropositivity data for each IIDD subcategory.

## Statistical analyses

The data were forwarded through the Internet and entered in an ad hoc Excel spreadsheet; analyses were performed using the Statistical Package for Social Science (SPSS), version 14. Characteristics were compared between categories and subcategories of IIDD using χ^2^ (or Fisher’s exact) tests for categorical data and two-sample *t*-tests for continuous data. When categorical variables were compared with continuous variables the Mann-Whitney U test was applied. Regression logistic models were applied to analyze association and risk. Geoprocessing resources were used to geographically visualize the information produced.

## Results

After 35 registries from patients without specific diagnoses were excluded, the data from 1,917 individuals with IIDD followed during 2011 across 22 reference centers for MS treatment, distributed across 17 cities in SA were analyzed. The reference centers that provided these data were located in four Spanish-speaking cities in SA and thirteen cities located across five Brazilian regions.

### The IIDD spectrum in SA

Based on the current diagnostic criteria, the following major categories and their frequencies were recorded with regard to the 1,917 patients with IIDD: MS (76.9%; 95% CIs = 75.0%-78.7%), NMO (11.8%; 95% CIs = 10.4–13.3), other NMOSDs (6.5%; 95% CIs = 5.5–7.7), CIS (3.5%; 95% CIs = 3.3–3.7), ADEM (0.99%; 95% CIs = 0.99–0.99), and acute IIDD with encephalopathy (0.37; 95% CIs = 0.34–0.39).

The frequency of all CNS IIDDs is described in Table 1.

**Table 1:** The IIDD spectrum in SA: The frequency of all categories and subcategories. IIDD = inflammatory idiopathic demyelinating disease; ADEM = acute disseminated encephalomyelitis; CIS = clinical isolated syndrome; MS = multiple sclerosis; RRMS = relapsing remitting at onset; PPMS = primary progressive; NMO = neuromyelitis optica; NMOSDs = NMO spectrum disorders; LETM = longitudinally extensive transverse myelitis.

Major diagnostic category	Diagnostic subcategory	N	% (95% CIs)
Acute IIDD with encephalopathy	Pseudotumor	4	0.21 (0.19–0.21)
N = 7 (0.37%; 95% CIs = 0.34–0.39)	Monophasic Balo’s concentric sclerosis	3	0.16 (0.14–0.17)
Acute Disseminated Encephalomyelitis (ADEM)	ADEM Monophasic	14	0.73 (0.71–0.75)
N = 19 (0.99%; 95% CIs = 0.99–0.99)	ADEM Polyphasic	5	0.26 (0.24–0.28)
CIS	CIS Optic Neuritis (ON)	33	1.7 (1.6–1.9)
N = 67 (3.5%; 95% CIs = 3.3–3.7)	CIS Brainstem (BS)	6	0.31 (0.29–0.33)
	CIS Transverse Myelitis	18	0.94 (0.93–0.95)
	CIS multifocal	10	0.52 (0.50–0.54)
Multiple sclerosis (MS)	RRMS	1,384	72.2 (70.2–74.2)
N = 1,474 (76.9%; 95% CIs = 75.0–78.7)	PPMS	90	4.7 (4.5–4.9)
Neuromyelitis optica (NMO)	NMO Monophasic	38	2.0 (1.8–2.2)
N = 226 (11.8%; 95% CIs = 10.4–13.3)	NMO Recurrent	188	9.8 (9.7–9.9)
Other NMOSDs	LETM monophasic	25	1.3 (1.2–1.5)
N = 226 (11.8%; 95% CIs = 10.4–13.3)	LETM recurrent	39	2.0 (1.9–2.2)
	LETM + BS	6	0.31 (2.9–3.3)
	Bilateral recurrent ON (BRON)	15	0.78 (0.76–0.80)
	ON + BS	1	0.05 (0.04–0.06)
	Optic spinal Asian type MS (OS-MS)	38	2.0 (1.8–2.2)
Total		1,917	100

The demographic data and the clinical evolution of the patients with IIDD are analyzed in Table 2.

**Table 2:** Demographic and clinical features of patients according to the major IIDD categories. MS = multiple sclerosis; NMO = neuromyelitis optica; NMOSD = NMO syndrome; CIS = clinical isolated syndrome; ADEM = acute disseminated encephalomyelitis; acute IIDD with encephalopathy; IIDD = inflammatory idiopathic demyelinating disease; EDSS **=** expanded disability status scale; MSSS **=** MS severity score

Variables		MS	NMO	Other NMO syndromes	CIS	ADEM	Acute IIDD with encephalopathy	All IIDD
		n = 1,474	n = 226	n = 124	n = 67	n = 19	n = 7	n = 1,917
**Gender**, N(%)	Female (F)	1,068 (72.5)	191 (84.5)	84 (67.7)	55 (82.0)	11(58.0)	5 (71.4)	1,414 (73.8)
	Male (M)	406 (27.5)	35 (15.5)	40 (32.3)	12 (18.0)	8 (42.9)	2 (28.6)	503 (26.2)
	Ratio F/M	2.6:1	5.4:1	2.1:1	4.6:1	1.4:1	2.5:1	2.8:1
**Skin color**, N(%)	White (W)	983 (66.7)	103 (45.6)	68 (54.8)	34 (50.7)	13 (68.0)	4 (57.0)	1,205 (63.0)
	Non-white (NW)	473 (32.1)	120 (53.1)	51 (41.12)	29 (43.3)	6 (32.0)	3 (43.0)	682 (35.2)
	Afro	387 (26.3)	89 (39.4)	42 (34.0)	21 (31.3)	3 (16)	2 (28.6)	544 (28.0)
	Mestizo	84 (5.7)	30 (13.3)	8 (6.5)	8 (11.9)	3 (16)	1 (14.3)	134 (7.0)
	Asian	2 (0.1)	1 (0.4)	1 (0.8)	0	0	0	4 (0.2)
	Ratio W/NW	2.1:1	0.86:1	1.3:1	1.2:1	2.1:1	1.3:1	1.7:1
	Missing	18 (1.2%)	3 (1.3%)	5 (4%)	4 (6%)	0	0	30 (1.6%)
**Age of onset**, N(%)	0–9 years	11 (0.7)	10 (4.4)	0	1 (1.5)	0	0	22 (1.1)
	10–19 years	155 (10.5)	36 (16.0)	17 (13.7)	12 (18.0)	5 (26.3)	1 (14.0)	226 (11.8)
	20–29 years	445 (30.2)	69 (30.5)	33 (26.6)	16 (24.0)	6 (31.6)	3 (43.0)	572 (29.8)
	30–39 years	441 (29.9)	48 (21.2)	33 (26.6)	20 (30.0)	2 (10.5)	3 (43.0)	547 (28.5)
	40–49 years	279 (18.9)	38 (16.8)	24 (19.4)	13 (19.0)	2 (10.5)	0	356 (18.6)
	50–59 years	111 (7.5)	22 (9.7)	13 (10.5)	0	1 (.3)	0	151 (7.9)
	60–69 years	16 (1.1)	3 (1.3)	3 (2.4)	4(6.0)	3 (15.8)	0	26(1.4)
	70–79 years	2 (0.1)	0	0	0	0	0	2 (0.1)
	Missing	14 (0.9%)	0	1 (0.8%)	0	0	0	15(0.8%)
	Mean±SD	32.9±11.3	31.2±13.56	34.1±12.7	31.4±12.1	32.5±0.81	27.4±7.0	32.7±11.8
**Clinical course**	RR: 1,384 (93.9)	M: 38 (16.8)		M:67	M: 14 (74.0)	M: 7		
	PP: 90 (6.1)	R: 188 (83.2)			O:5 (26.0)			
**Time of disease**	Mean+SD	9.78 ± 7.86	8.91 ± 6.89	7.42 ± 7.60	3.54 ± 3.51	9.11 ±11.92	4.43 ±6.02	9.28±7.76
	Missing	5 (0.3%)	0	1 (0.8%)	0	0	0	6 (0.3%)
EDSS	Median (min-max)	25 (0–0.5)	4 (0–9.5)	3 (0–8,5)	1 (0–7)	3 (0–9)	1 (1–3)	
	EDSS mild	814 (55.2%)	58 (25.7%)	47 (37.9%)	51 (77.3%)	9 (47.4%)	6 (85.7%)	
	**EDSS moderate**	369 (25.0%)	93 (41.2%)	39 (31.5%)	12 (18.2%)	2 (10.5%)	1 (14.3%)	516 (26.9%)
	**EDSS severe**	283 (19.2%)	73 (32.3%)	38 (30.6%)	3 (4.5%)	8 (42.1%)	0	404 (21.1%)
	**Missing**	8 (0.5%)	2 (0.9%)	1 (1.5%)	1 (1.5%)	0	0	12 (0.6¨%)

Females predominated all six major categories, ranging from 58.0% of the ADEM group to 84.5% of the NMO group. Whites also predominated the six categories, except for the NMO group in which 53.1% of the affected individuals were non-white. Only four cases of IIDD (two cases of MS, one case of NMO, and one case of other NMOSDs) were reported in Asians; these patients lived in São Paulo (two cases), Brasília, or Buenos Aires (one case each). No native Indians were affected. The mean age at disease onset was 32.7±11.8 years. The majority of the patients in all of the major categories, except ADEM, started exhibiting symptoms between 20 and 39 years old (ranging from 51.1% of those with NMO to 86.0% of those with acute IIDD with encephalopathy). Early onset occurred in 8.2% of all patients (0% of those with acute IIDD with encephalopathy, 7.0% of those with MS, 7.3% of those with NMOSD, 10.4% of those with CIS, 15.0% of those with NMO, and 21.1% of those with ADEM); late onset occurred in 8.9% (0% of those with acute IIDD with encephalopathy, 7.5% of those with CIS, 9.4% of those with MS, 11.5% of those with NMO, 12.9% of those with other NMOSDs, and 21.1% of those with ADEM). The long-term morbidity after a mean disease time of 9.28±7.7 years (as analyzed by the EDSS) was characterized by mild disability in the total population. All categories except for NMO were scored as moderate. In the MS category, disease time was positively correlated with the EDSS score (r = 0.374; p = <0.001). This correlation was not observed among those with NMO (r = 0.129; p = 0.054) or NMOSDs (r = 0.22; p = 0.812). With respect to the diagnostic tests, more than 98% of the individuals with IIDDs underwent brain and spinal cord MRIs; CSF analyses were performed in more than 90%, and VEP was performed in more than 80%. NMO-IgG antibody was tested for in 324 patients (acute IIDD with encephalopathy [n = 2], ADEM [n = 2], CIS [n = 17], MS [n = 87], NMO [n = 162], and other NMOSDs [n = 54]). All but one neurological center used the IIF to test the NMO-IgG antibody (95%); the Elisa method was applied in the remaining center.

### IIDD phenotypes

[1] Rare and acute forms—Acute forms of IIDD with encephalopathy were diagnosed using brain MRI and confirmed by brain biopsy in seven patients. Unique pseudotumoral brain lesions were identified in three white patients and one white Mestizo (two cases in Rio de Janeiro, one case in Maracaibo, and another in Asuncion); the mean age of these patients at onset was 29±6.9 years. The follow-up assessments of these patients, which lasted from 1 to 18 years (mean = 5.75±8.18 years), did not reveal other neurological manifestations, maintaining a mild disability score according to the EDSS. The diagnosis of Balo in vivo was established in one white and two Africans individuals (mean age = 25.33±7.76 years; São Paulo) who showed mild disability after 2.67±0.57 years of follow up.

[2] ADEM (monophasic and recurrent)—ADEM monophasic was recorded in 14 patients among whom the only acute event occurred at a mean age of 36.64±19.87 years, ranging from younger than 18 (21.4%) to older than 50 years old (28.7%). Most of these patients showed mild disability according to the EDSS. Only five cases of recurrent ADEM were recorded; these cases had earlier onsets and worse long-term disability.

[3] CIS—Acute forms of demyelinating inflammatory syndromes suggestive of MS were recorded in 67 patients, mostly women, with an age of onset between 20 and 39 years old (54.0%). Onset before 18 years old occurred in 10.4% of cases and after 50 years old occurred in 7.5% of cases.

Four classes of CIS were defined. The most frequent was optic neuritis (49.3%), followed by transverse myelitis (26.9%), multifocal CIS (14.9%), and brainstem syndrome (BS) (9.0%). At the last follow up, after a mean disease time of 3.54±3.51 years (minimum 1 year, maximum 18 years), the disability was mild for all clinical presentations.

[4] MS—Data from 1,474 patients with MS, of whom 1,384 (93.9%) had RRMS and 90 (6.1%) had PPMS since onset, were analyzed. Approximately 73.0% of the patients with RRMS were women, and 66.6% white. The disease onset occurred between 20 and 39 years old in 60.7% of patients; 7.2% included pediatric forms (0.8% up to 9 years old), and 8.5% included cases of late onset. The majority of the patients had mild long-term disability (58.3%). The others showed moderate (24.9%) or severe disability (16.3%). Secondary progression was observed in 14.6% (203/1,384) of cases. Approximately 63.0% of the patients with PPMS were women, and 67.8% white. The disease onset occurred between 30 and 49 years old (57.7%), and 22.2% of the cases were late onset. At the last follow up, the majority of patients with PPMS showed severe disability (64.4%); the others showed moderate (26.7%) or mild disability (7.8%).

To test the possible factors associated with disease severity (i.e., EDSS scores ≥6), a multinomial model was applied that considered the following variables: clinical course at onset (PP vs. RR), gender, age at onset (> or < the median), ethnicity (non-white vs. white), early onset forms, late onset forms, latitude of the reference center in SA (> or < the median), and disease time (> or < the mean). The PP clinical course and longer times of disease were associated with greater disability. Applying the same model to the RRMS and PPMS groups separately, disease duration remained significant in both subcategories. The risk for secondary progression was significantly higher in Africans (HR = 1.46, p = 0.021). Because the time of MS influences the severity of the disability, we applied the MSSS score. Using this index with regard to the RRMS group, long-term severity was classified as mild-moderate for the majority of patients (56.4%), followed by advanced-accelerated (25.5%), benign (9.4%), and aggressive-malignant (8.7%). Most of the patients with PPMS were included in the aggressive-malignant (48.0%) or advanced-accelerated (34.8%) groups; the others had the mild-moderate form (16.9%).

[5] NMO—A total of 226 patients met the criteria for definite NMO (2006), of whom 188 (83.2%) had the relapsing-remitting course, and 38 (16.8%) had the monophasic course. Women and Africans predominated the recurrent NMO group. The majority (55.8%) presented with their first acute event between 20 and 39 years old; pediatric forms of NMO (early onset) occurred in 14.9%, late onset occurred in 8.0%, and the long-term disability was moderate (44.1%) or severe (34.0%). Women and whites predominated the monophasic NMO group; pediatric forms (early onset) comprised 15.8% of this group; 31.6% of patients stated that the disease began between 20 and 39 years old, 23.7% stated that the disease began between 40 and 49 years old, and 26.3% stated that the disease began after 50 years old. The long-term disability was moderate (26.3%) or severe (23.7%). Comparing these variables with regard to clinical course, recurring NMO differs from monophasic NMO by the greater frequency of Africans (p = 0.022), earlier age of onset (p = 0.009), and higher disability (p = 0.014).

[6] NMOSDs—***[6*.*1] Limited NMO syndromes (n = 86)*:** LETM was documented in 70 of 86 cases (81.4%). The recurrent LETM (n = 39) was predominated by women and Africans. The first TM acute event occurred between 30 and 49 years old (46.2%); 17.9% had late onset, and 7.7% had early onset. Most patients developed moderate or severe disability (69.2%). The monophasic LETM group (n = 25) was predominated by white men who had an episode of myelitis between 30 and 49 years old (60.0%) and developed severe disability (64.0%). Comparing these variables with regard to the clinical course, more women had recurring LETM (p = 0.004), and neurological disability was more likely in monophasic LETM (p = 0.011). Six patients, most of them women and African, had LETM an associated BS event with mild (42.9%) or severe (42.9%) disability at the last follow up. BRON occurred in 15 patients; most were white women for whom the disease began at 10 to 19 years old (20.0%), 20 to 29 years old (26.7%), or 30 to 39 years old (20.0%). The long-term disability assessed by the EDSS was mild (66.7%). Only one patient had bilateral ON with a BS event.

The demographic, clinical, and laboratorial data of the patients with NMO and limited syndromes are shown in Table 3.

**Table 3:** Demographic, clinical, and laboratory characteristics with regard to NMOSD subcategories. Differences in totals are due to missing values. Legend. LETM, Longitudinally extensive transverse myelitis; BRON, bilateral recurrent optic neuritis; BS, brainstem syndrome; NMOSD, NMO syndrome; MSRR, relapsing-remitting multiple sclerosis; MSSP, multiple sclerosis secondary progressive

Variables		NMO		Limited NMOSD syndromes			
		NOM M (n = 38)	NMOR n = 188	ONRB n = 15	LETMM n = 25	LETMR n = 39	LETM or ON +BS n = 7
**Gender**, N(%)	Female (F)	30 (78.9%)	161 (85.6%)	12 (80%)	9(36%)	29 (74.4%)	5 (71.4%)
	Male (M)	8 (21.1%)	27 (14.4%)	3 (20%)	16(64%)	10 (25.6%)	2 (28.6%)
	White (W)	22 (57.9%)	81 (43.1%)	10 (66.7%)	15(60%)	14 (35.9%)	3 (42.9%)
**Skin color**, N(%)	Afro	8 (21.1%)	81 (43.1%)	2 (13%)	7 (28%)	17 (43.6%)	4 (57.1%)
	Mestizo	8 (21.1%)	22 (11.7%)	3 (20%)	1 (4%)	4 (10.3%)	0
	Asian	0	1 (0.5%)	0	1 (4%)	0	0
	Missing	0	3 (1.6%)	0	1 (4%)	4 (10.3%)	0
**Age at onset**, N(%)	0–9 years	1 (2.6%)	9 (4.8%)	0	0	0	0
	10–19 years	6 (15.8%)	30 (16%)	3 (20%)	3 (12%)	5 (12.8%)	1 (14.3%)
	20–29 years	5 (13.2%)	64 (34%)	4 (26.7%)	5 (20%)	8 (20.5%)	1 (14.3%)
	30–39 years	7 (18.4%)	41 (21.8%)	3 (20%)	7 (28%)	9 (23.1%)	2 (28.6%)
	40–49 years	9 (23.7%)	29 (15.4%)	3 (20%)	8 (32%)	9 (23.1%)	0
	50–59 years	9 (23.7%)	13 (6.9%)	2 (13.3%)	1 (4%)	5 (12.8%)	3 (42.9%)
	60–69 years	1 (2.6%)	2 (1.1%)	0	1 (4%)	2 (5.1%)	0
	Missing	0	0	0	0	1 (2.6%)	0
	Age ≤17	6 (15.8%)	28 (14.9%)	2 (13.3%)	2 (8%)	3 (7.7%)	0
	Age ≥50	10 (26.3%)	15 (8%)	2 (13.3%)	2(8%)	7 (17.9%)	3 (42.9%)
**Age of onset**	Mean+SD	37.02±15.33	30.06±12.9	32.73±13.05	36.16±11.68	36.47 ±14.24	37.14 ±14.42
**Time of disease**	Mean+SD	8.47±6.97	9±6.89	4.53±6.25	4.88±6.88	6.89±7.42	7±5.16
	Median (min-max)	3 (0–8)	4 (0–9.5)	2.5 (1–4)	6 (0–8.5)	4 (0–8)	4 (0–7)
	**EDSS mild**	18 (47.4%)	40 (21.3%)	10 (66.7%)	2 (8.0%)	11 (28.2%)	3 (42.9%)
	**EDSS moderate**	10 (26.3%)	83 (44.1%)	5 (33.3%)	7 (28%)	14 (35.9%)	1 (14.3%)
	**EDSS severe**	9 (23.7%)	64 (34%)	1 (6.7%)	16 (64%)	13 (33.3%)	3 (42.9%)
	**Missing**	1 (2.6%)	1 (0.5%)	0	0	1 (2.6%)	0
**AQP4+**		14/25 (56.0%)	81/137 (59.1%)	0	1 (4%)	4 (10.3%)	0

***[6*.*2] OS-MS (Asian-type) in SA-*** A total of 38 patients were classified as OS-MS with acute and recurrent events restricted to the optic nerve and spinal cord with a mean disease time of 10.84±8.10 years old. Whites (68.4%) and females (73.3%) predominated. The first event was diagnosed at 20 to 39 years old (71.1%); 5.3% showed early onset, and the same frequency showed late onset. Long-term disability assessed using the EDSS was mild (55.3%), moderate (31.6%) or severe (13.2%).

NMO-IgG antibody positivity with regard to the subcategories

The NMO-IgG antibody was tested in 324 patients with IIDD. Seronegativity was found in one patient with a large cerebral lesion, one case of Balo disease, two cases of ADEM, 17 cases of CIS, 16 cases of OS-MS, and all but one case of RRMS (86/87). The antibody was positive in patients with recurrent NMO (81/137; 59.1%), monophasic NMO (14/25; 56.0%), recurrent LETM (6/17; 35.3%), monophasic LETM (3/9; 33.3%), LETM or ON with BS (1/4; 25%), and in all cases of BRON (8/8; 100%).

We analyzed the influence of NMO-IgG antibody positivity on the 200 patients with NMOSDs (NMO: n = 162; LETM: n = 30; BRON: n = 8). NMO-IgG was positive in 56.5% of all cases. No associations were found with regard to gender, age at onset (< or > the median), ethnicity, early or late onset, disease course (monophasic or recurrent), reference center latitude (< or > the median [-22°S]), and long-term severe disability (i.e., EDSS = 6).

The frequencies of IIDDs in SA by ethnicity

**Fig 1:**
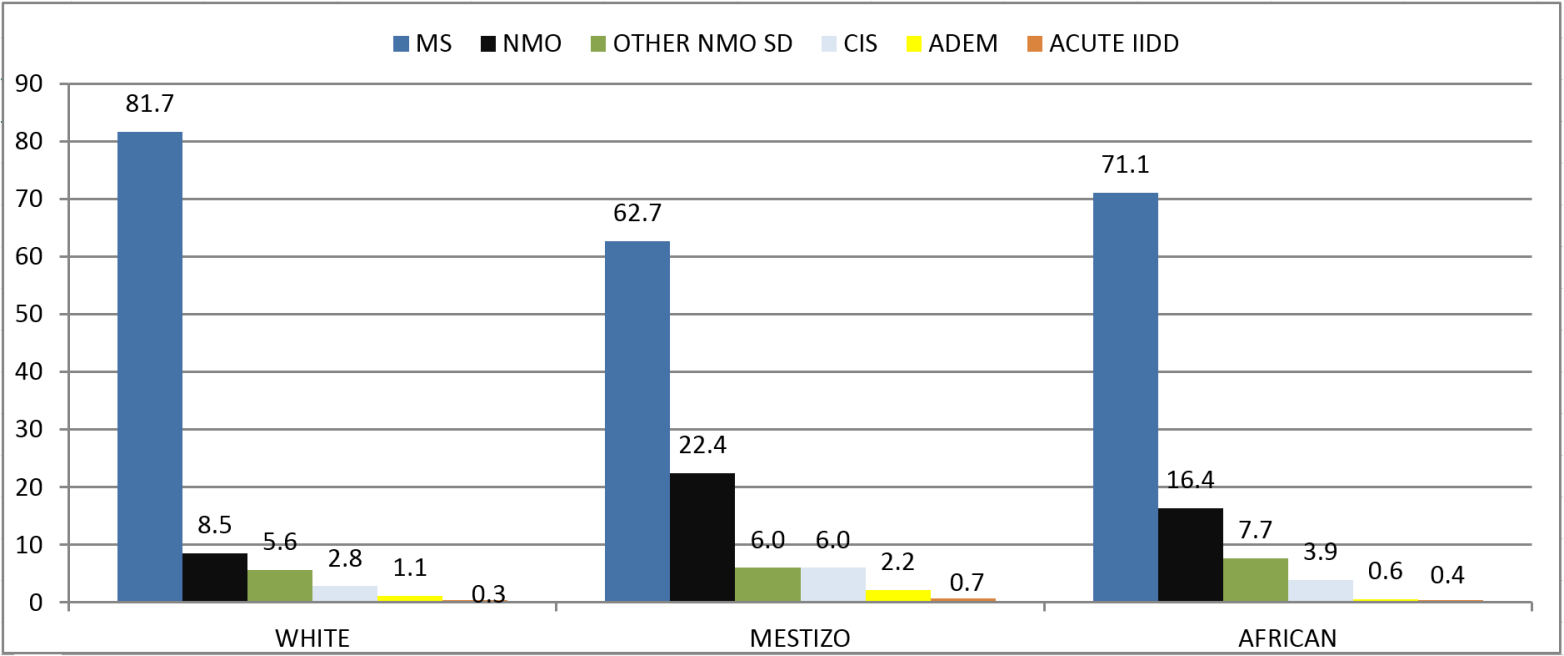
The IIDD spectrum in SA by ethnicity. IIDD = inflammatory idiopathic demyelinating disease, MS = multiple sclerosis; NMO = neuromyelitis optica; NMOSD = NMO syndrome; CIS = clinical isolated syndrome; ADEM = acute disseminated encephalomyelitis; acute IIDD with encephalopathy;

Fig 1 shows the distribution of the six major categories of IIDD by ethnicity.

MS was the most common IIDD, followed by NMO among whites, Mestizos, and Africans. However, a significant difference was observed when comparing the distribution of those six categories among whites versus mestizos (p<0.001) and white versus Africans (p<0.001). A difference was not found between Mestizos and Africans (p = 0.12).

NMO versus RRMS

Diagnoses of NMO differed from those of RRMS at onset by demographic, clinical, and laboratory characteristics (see Table 4).

**Table 4:** NMO versus RRMS. MSRR = multiple sclerosis relapsing remitting; MSPS–multiple sclerosis secondary progressive; NMO = neuromyelitis optica.

Variables		MSRR+PS, n = 1384	NMO, n = 226	p-value
Gender, N(%)	**Female (F)**	1,011 (73,0%)	191 (84.5%)	<0.001
	**Male (M)**	373 (27.0%)	35 (15.5%)	
Skin color, N(%)	**White (W)**	922 (66.6%)	103 (45.6%)	<0.001
	**Afro**	362 (26.2%)	89 (39.4%)	<0.001
	**Mestizo**	82 (5.9%)	30 (13.3%)	<0.001
	**Asian**	2 (0.1%)	1 (0.4%)	
	**Missing**	16 (1.2%)	3 (1.3%)	
Age at onset, N (%)	**1st decade**	11 (0.8%)	10 (4.4%)	<0.001
	**2nd decade**	152 (11.0%)	36 (15.9%)	0.038
	**3rd decade**	430 (31.1%)	69 (30.5%)	0.33
	**4th decade**	410 (29.6%)	48 (21.2%)	0.010
	**5th decade**	258 (18.6%)	38 (16.8%)	0.26
	**6th decade**	99 (7.2%)	22 (9.7%)	0.10
	**7th decade**	11 (0.8%)	3 (1.3%)	0.25
	**8th decade**	0	10 (4.4%)	0.23
	**Missing**	13 (0.9%)	0	
Age at onset (years)	**Mean ± SD**	32 ± 11 (4.0–66.0)	31.2 ± 13.5 (2.0–68.0)	0.14
Disease time (years)	**Mean ± SD**	9.6 ± 7,7 (1–47)	8.9 ± 6.89 (1–38)	0.19
Disability	**Median (min-max)**	1.0 (1.0–4.0)	4.0 (0–9.5)	<0.001
	**EDSS mild**	807 (58.3%)	58 (25.7%)	<0.001
	**EDSS moderate**	345 (24.9%)	93 (41.2%)	<0.001
	**EDSS severe**	225 (16.3%)	73 (32.3%)	<0.001
	**Missing**	7 (0.5%)	2 (0.9%)	

Approximately 1.0% of patients with NMO+RRMS in Argentina were non-white (Asian = 1); 30.4% of these patients in Paraguay were non-white (Mestizos = 42); 36.8% of these patients in Brazil were non-white (Mestizos = 13 and Africans = 483), and 79.1% of these patients in Venezuela were non-white (Mestizos = 50 and Africans = 10). These frequencies were 9.6%, 37.5% 36.5%, 96.8%, and 60.9% in the southern, southeastern, midwestern, northeastern, and northern regions of Brazil. The relative frequency of NMO among NMO+RRMS cases in SA was 14.0%: 43.3% in Venezuela, 14.0% in Brazil, 8.7% in Paraguay, and 2.1% in Argentina.

Table 5 displays the relative frequency of NMO cases across the 22 MS centers located in 18 cities in SA. The most southern latitudes were generally associated with fewer NMO cases.

**Table 5:** The relative frequencies of NMO among patients with NMO+RRMS at onset by latitude. RRMS = relapsing remitting multiple sclerosis at onset; NMO = neuromyelitis optica.

MS location center	Latitude	Ethnicity	Frequency of NMO among NMO + RRMS		
		Frequency of non-whites		Brazilian regions	Country
Caracas	10°N	79.1%	43.3%	__	Venezuela (43.3%)
Belém (1)	1°S	41.6%	12.5%	North (15.2%)	Brazil (14.0%)
Belém (2)	1°S	66.7%	16.6%		
Recife	8°S	96.8%	3.2%	Northeast (3.2%)	
Brasília	15°S	37.1.%	18.8%		
Cuiabá (1)	15°S	50.0%	37.5%	Midwest (11.6%)	
Cuiabá (2)	15°S	30.4%	8.9%		
Goiania	16°S	39.0%	1.3%		
Belo Horizonte	19°S	56.0%	20.5%	Southeast (17.9%)	
Rio Janeiro (1)	22°S	38.3%	10.4%		
Rio Janeiro (2)	22°S	11.9%	4.4%		
Rio Janeiro (3)	22°S	27.9%	16.3%		
Rio de Janeiro-Sul Fluminense	22°S	30.6%	18.3%		
Santos	23°S	16.1%	8.9%		
São Paulo	23°S	41.7%	38.1%		
Curitiba	25°S	4.2%	4.1%	South (5.1%)	
Joinville	26°S	1.3%	7.7%		
Florianópolis	27°S	17.9%	3.1%		
Assuncion (1)	25°S	12.5%	25.0%	__	Paraguay (8.7%)
Assuncion (2)	25°S	32.8%	6.5%	__	
Buenos Aires	34°S	1.0%	2.1%	__	Argentina (2.1%)

## Discussion

Epidemiological studies have found low to medium rates of MS (1.48 a 17.1/100.000) in SA, with values increasing along a north-south gradient below the equator. Conversely, these rates vary from medium to high along a south-north gradient in North America (NA). These differences can be explained by the genetic composition of the population and by geographical position, which might favor environmental factors such as sun exposure [26–28]. Although the prevalence of MS in SA is less than that in NA and Europe, the results presented in this multicenter study demonstrate that demographic and clinical characteristics have comparable patterns to those in high-prevalence areas. MS was the most frequent IIDD, primarily affecting white women. The most common age at onset occurred between the second and fourth decades. The clinical course was relapsing-remitting at onset in approximately 94% of all cases; this course was associated with less disability than the primary progressive course over time [29–31]. Optic neuritis, transverse myelitis, and BS were the main CISs, as recently described [21]. Early onset (i.e., pediatric) MS cases were slightly more frequent in SA (7.3%) than Europe and the US (3%-5.5%) [32]; all of these cases showed an initial relapsing-remitting presentation, and most patients were white females who had their first relapse after 10 years old (90%). Disease onset before 10 years old was rare (0.8%).

Knowledge of the epidemiology and natural history of MS is crucial for physicians and patients to make informed decisions regarding their care [2]. The mild disability according to the EDSS among 58.3% of the patients with RRMS after an average of almost 10 years (and the fact that only 14.6% of those cases showed secondary progression) suggests the presence of a more benign course of MS in SA. However, this suggestion can only be confirmed via a prospective follow up of this cohort that accounts for confounds such as the use of disease-modifying therapy (DMT). Despite the social contrast in SA, DMT was available for patients with MS at public healthcare centers in Brazil, Argentina, and Venezuela over the last 15 years. Prognostic studies of MS in SA are rare; only three longitudinal studies [33–35] have investigated the prognostic factors associated with disability and progression in Brazil, concluding that the number of relapses in the early years, the time interval between the first and the second relapses, an older age at onset, and the male gender influenced disease progression. These findings are similar to those of studies conducted in NA and Europe [36–37]. Population studies on the progression of MS disability are difficult to perform because the relationship between the EDSS and disease duration is not linear because of the considerable individual variation in the clinical course of this disease. In the present study, the severity of disability at last assessment was associated with disease time and a progressive clinical course. When we applied the MSSS index [24] (a proposed epidemiological method featuring a single evaluation of EDSS adjusted by disease time), we observed that the pattern of disease progression in MS was broader than the disability measured using the EDSS. The long-term severe disability observed in patients with RRMS was classified as mild-moderate (56.4%), whereas most patients with PPMS were categorized as aggressive-malignant (48.0%) [25]. We found that Africans were at major risk for secondary progression, a link previously described among African-Americans [38] and African-Brazilians [34–35].

Unlike MS, NMO is a rare disease worldwide with estimated prevalence of 0.52/100,000 in Cuba (according to a 2009 nationwide, non-laboratory survey that analyzed 11.2 million people, featuring a mixture of African-Cubans and Spanish; two specialists performed the clinical testing at one center); 4.2/100,000 in the French Antilles (i.e., Martinique and Guadalupe, according to a 2009 population study that examined 645,000 people, of whom 73% had African ancestry; the serum AQP4 antibody was not tested); 4.4/100,000 in the southern region of Denmark (according to a 2011 study of 952,000 adults, of whom 94.1% were ethnic Danes; the serum AQP4 antibody was tested); 1.9/100,000 in southeast Wales (according to a 2012 study of 712,572 people, of whom 95.6% were white; the serum AQP4 antibody was tested); 0.72/100,000 in Merseyside, United Kingdom (according to a 2013 study of 1.1 million adults; the AQP4 antibody was tested in a central lab); and 0.71/100,000 in Austria (according to a 2013 nationwide survey of 8.4 million people, all white; the AQP4 antibody was tested in a central lab) [39–43].

NMO comprised 11.8% of all cases of IIDD in SA, affecting mostly young African-Brazilian women, evolving with a recurrent course after the index events (ON/TM) and causing moderate or severe disability. Except for ethnicity, these data are similar to the NMO series diagnosed among whites using the 2006 criteria. [44–45] Early onset (i.e., pediatric forms) occurred in 15.0% of patients, and more than a quarter of these patients had their first acute event when they were younger than 10 years old. No differences in gender, ethnicity/skin color, or morbidity were found between patients younger and older than 18 years old. NMO differs significantly from MS with respect to gender, ethnicity, and disability as previous studies in southeastern Brazil have demonstrated [15,46]. Ethnicity did not affect NMO-related long-term disability. NMO-positive antibodies were associated with worse prognoses in African-Caribbean [47]; however, the presence of this antibody was not associated with higher morbidity (EDSS score ≤6), regardless of the ethnic background among patients with NMO from SA.

In SA, the NMO-IgG antibody positivity in patients with NMO tested using the IIF method [8] was slightly higher among Africans (44/71; 62.0%) than whites (40/71; 56.3%) or Mestizos (7/16; 40.3%). These results did not confirm those of a prior study that described a lower positivity rate (16/48; 33%) in the Caribbean where the population was predominantly of African descent [47]. Approximately 73% (33/45) of American patients were positive in Lennon's [8] original report; however, other NMO series that applying the IIF found frequencies of 61.1% (22/36), 62.5% (18/28), 54% (14/26 and 60/111), and 39.4% (13/33) in Germany [48], Spain [49], France [45, 50], and Italy [51], respectively. The NMO-IgG positivity observed in patients with NMO from São Paulo was similar to that in those from Europe: 64.3% (18/28) [52] and 41% (7/17) [53].

Other methods of NMO-IgG detection were developed to increase test sensitivity. Takahashi and colleagues [54] identified human cell lines that expressed AQP4 after being transfected and used them as a substrate of IIF to detect anti-AQP4 antibody. Paul et al. and Waters et al. used tests with recombinant human AQP4 (RIPA/FIPA) [55, 56]. Hayakawa and colleagues developed the method for detecting anti-AQP4 enzyme-linked immunosorbent assay (ELISA) using AQP4 antigen extracted from mice [57]. An international, collaborative comparison of the sensitivities of those assay methodologies confirmed that the IIF assay was less sensitive than the second-generation recombinant antigen-based assays [58]. These studies opened the discussion regarding NMO-IgG and AQP4-serum autoantibody detection methods and the inclusion criteria of epidemiological studies. Assay insensitivity might overestimate the frequency of seronegativity. Jarius and colleagues [59] analyzed the influence of antibodies on the clinical course of NMOSDs in whites (119 with NMO, 49 with isolated LETM, and 7 with recurrent ON). The majority of the cases (78.3%) tested AQP4-antibody positive using a cell-based assay. Seropositive patients were predominantly female, and the disease course was more often monophasic in seronegative patients; seropositive and seronegative patients did not significantly differ with regard to age at onset or annual EDSS increase. Jiao and colleagues [60] tested the available stored sera of 49 NMO “seronegative” patients at the Mayo Clinic using new recombinant antigen-based assays and reclassified 30 patients (61%) as seropositive. Seronegative patients differed from seropositive patients by sex ratio (> male) and onset attack (simultaneous ON and TM). Relapse rate, disability outcomes, and other characteristics did not differ between groups. Marignier and colleagues performed a similar study in France [61] using the IIF method and cell-based assays. They demonstrated a direct relationship between the increasing sensitivity of the antibody detection method and identified the characteristics of the seronegative group, such as a lower female:male ratio, fewer whites, and an overrepresentation of simultaneous optic neuritis and transverse myelitis at onset. The current study compared gender, ethnicity, age at disease onset, disease course, and disability according to NMO-IgG status in 200 NMOSD patients, and no significant differences were found. The present study was limited by the use of IIF in 95% of the MS centers in SA. IIF is inferior to highly sensitive and specific cell-based immunoassays.

Monophasic and recurrent acute transverse myelitis (ATM) [62] and chronic recurrent idiopathic optic neuritis (CRION) [63] were recognized as IIDDs prior to the identification of NMO IgG by Lennon et al. (2004) [8]. These conditions were included as NMOSDs, considering their clinical and neuroimaging similarities with NMO and their (albeit less frequent) positivity for anti-IgG NMO [22]. The presence of NMO-IgG in patients with limited NMO syndromes such as LETM and BRON was associated with an increased risk of conversion to NMO [8] and recurrence [64].

LETM represents the second most frequent phenotype of NMOSD in SA. In its monophasic presentation, men most likely to be affected, and they developed severe and rapid disability after a single myelitis, whereas women were more likely to be affected by recurrent LETM with moderate disability (according to the EDSS) after multiple events of myelitis. NMO-IgG antibody was positive in approximately one-third of the cases tested. No association was found between the presence of the antibody and disability severity. In the original study, the frequency of NMO-IgG antibody in patients with LETM was 52% (14/27) [8]; other series of white participants with fewer of cases varied including rates of 80% (4/5) [48], 54% (7/13) [50], 50% (5/11) [49] and 15.4%-38.5% (12 LETM + 1 BRON) in a study that analyzed the positivity of the antibody using five different assays [51]. One Brazilian study found an antibody positivity of 41.2% (7/17) in patients with recurrent MTA and extensive spinal cord lesions among whom the presence of the antibody was not associated with greater disability [65]. The current study recorded only 15 patients with BRON; NMO-IgG positivity was found in all of the tested cases (100%; 8/8); conversely, a previous series found a much smaller antibody frequency in patients with recurrent optic neuritis than those with LETM, ranging from 25% (2/8) [8] to 14.3% (1/7) [49].

Asian type OS-MS is a common phenotype among the Japanese, and similarities between this syndrome and neuromyelitis had already been recognized by the early 2000s [17]. OS-MS was included as an NMOSD [22] after the NMO-IgG antibody was identified in the serum of Japanese patients (54%; 6/11) diagnosed with OS-MS [8]. Subsequent research conducted in Japan showed that NMO-IgG antibody positivity was only confirmed in OS-MS patients with LETM (61.5%; 16/26); patients with OS-MS without LETM and patients with Western-type MS were negative for this antibody, suggesting that Japanese patients with OS-MS and extensive spinal cord lesions have an underlying pathogenesis common to NMO [66,67]. In SA, the OS-MS phenotype differs from the other NMOSDs including NMO-IgG antibody negativity for all of the cases tested; young white women were most affected and exhibited mild disability after 11 years of disease. These clinical and laboratory features indicate that OS-MS is an RRMS phenotype in our population. If the 38 OS-MS cases were included in the MS group, then they would represent 2.6% of all RRMS cases (38/1,422). These results differ from one American study that found that OS-MS comprised 8.8% of 1,290 MS cases (125/1290), with a higher frequency among African-Americans; 8.8% (11/125) tested positive for the NMO-IgG antibody. Certain limitations of this study include the recruitment of patients with MS after excluding NMO cases that applied older NMO criteria (1999) and the arbitrary OS-MS definition based on the signs and symptoms restricted to the optic nerves and spinal cords of patients with at least 5 years of living with the disease [38].

The results of this study, which was conducted across different regions of SA, confirmed the influence of ethnicity on the frequency of NMO. The relative frequency of NMO among patients with NMO+RRMS was 14.0%, rising in a south-north gradient, from 2.1% at 34°S to 43.3% at 10°N (just above the Equator; see Table 5). Similarly, we observed the same gradient for the proportion of non-white patients. Argentina, which received significant European colonization and which has few African inhabitants, showed a relative NMO frequency of 2.1% among patients with MS. This rate is close to the frequency reported in studies conducted among whites living in Australia and Italy [12–13]. In Paraguay, where 30% of the patients were Mestizos, the relative frequency of NMO was higher (8.7%), which is similar to Mexico City (8%) where 60% of population is Mestizo [14].

Starting in south Brazil (which has a strong history of German and Italian colonization) with 5.1% of all NMO cases and moving north toward the equator, this territory corresponded to a fairly large area inhabited by individuals of both European and African ancestry as well as natives; furthermore, an increasing frequency of NMO was found. These results confirmed the south-north gradient in which Caracas and Maracaibo exhibited the highest frequency of NMO cases (43.3%). The exception to this pattern was found in the northeast, where only two cases of NMO were diagnosed despite the higher frequency in non-white patients (96%). A study will be required in Salvador (Bahia), São Luiz (Maranhão), and Fortaleza (Ceará) to determine the real frequency of NMO in this region.

The ethnic and socioeconomic characteristics of individuals with NMO, most of whom have African ancestry and low incomes, and the type of medical care provided at the investigated reference centers, might also be associated with differences in the frequency of NMO found within a single geographical area. For instance, striking differences were found in Rio de Janeiro between a public and a private MS reference center, even though both were operated by the same neurological staff. As another example, the frequency of NMO cases was 41.0% at Santa Marcelina Hospital, a large public hospital in São Paulo that admits underprivileged patients, whereas the average frequency of NMO found by a previous study at three MS reference centers in São Paulo was 6%. [16]

This study is limited by the lack of a method regarding ethnicity/skin color classification. Although the terms white, African, and Latino are used in epidemiological studies worldwide, the large miscegenation that characterizes the SA population suggests that ethnic and skin color groups are not homogeneous. A Brazilian study investigated the genetic contribution of European, African, and Amerindian ancestry among patients with MS or NMO, most of whom were from São Paulo, and it found that the contribution of European ancestry was higher in patients with MS than those with NMO, whereas African ancestry was higher in patients with NMO than those with MS. Moreover, a principal component analysis showed that patients with NMO from the south Brazil were clustered close to the European ancestral population [68].

Another relevant issue concerns the locations where the patients were selected (i.e., exclusively in treatment MS centers), which might underestimate the frequency of monophasic cases in which, after a single initial acute event, remission occurs with mild disability or even without disability and no indication for regular medical treatment. The monophasic ADEM represented in this study comprised less than 1% of all IIDDs; it affected patients during different decades of life, none of whom were younger than 10 years old, even though this syndrome is more frequent in childhood.

The identification of a high frequency of NMOSD cases has many implications for healthcare services in SA. NMO and LETM are severe conditions characterized by high morbidity in the short term; thus, they demand early diagnosis and the immediate institution of specific treatments for acute attacks (e.g., intravenous corticosteroids, plasmapheresis, or human immunoglobulin IV). Individuals with NMOSD are frequently admitted to neurological or intensive care units because of the risk of respiratory failure and the severity of their neurological abnormalities. The NMO-IgG testing applied to all suspected cases of IIDD will certainly help the early diagnosis of NMOSD. Finally, the rights endowed to patients with MS should also be extended to patients with NMO, including access to off-label drug use. Although the FDA has not yet approved medication for this condition, certain drugs have been potentially beneficial in case series [69]; however, healthcare services usually refuse to provide them.

## Conclusions

Despite the ethnic diversity of its population, all IIDD phenotypes in SA were found. Three-quarters of these patients had MS, which primarily affects white women and young people, showing similar clinical features to those in Western populations in the northern hemisphere. NMO primarily affects Africans, 58.6% of whom tested positive for NMO-IgG antibody (95% of whom were tested using IIF). NMO causes moderate/severe disability in both whites and Africans. The antibody positivity shown with regard to NMO was slightly higher among African descendants than whites. The presence of this antibody was not associated with higher long-term disability, regardless of ethnic background. Asian-type OS-MS presents demographic, clinical, and laboratory characteristics of MS in SA, including a predominance in whites, a favorable long-term prognosis, and the absence of NMO-IgG antibody, which suggests that this condition is a phenotypic subcategory of MS and not an NMOSD. The south-north gradient that shows increasing NMO among non-white individuals from Argentina, Paraguay, Brazil, and Venezuela confirmed the results of previous studies showing a higher frequency of NMO among non-white populations living in areas with a low prevalence of MS.

## Supporting Information

S1 File Demographic and clinical spreadsheet data from 1,917 IIDD patients. (ZIP)
